# Economic evaluation of surgical insertion of ventilation tubes for the management of persistent bilateral otitis media with effusion in children

**DOI:** 10.1186/1472-6963-14-253

**Published:** 2014-06-13

**Authors:** Syed Mohiuddin, Anne Schilder, Iain Bruce

**Affiliations:** 1 Manchester Centre for Health Economics, Institute of Population Health, University of Manchester, Oxford Road, Manchester M13 9PL, UK; 2 UCL Ear, Nose and Throat Clinical Trials Programme, University College London, Gower Street, London WC1E 6BT, UK; 3 Paediatric ENT Department, Royal Manchester Children’s Hospital, Manchester M13 9WL, UK

**Keywords:** Otitis media with effusion, Children, Ventilation tubes, Economic evaluation, Hearing aids, Cost-effectiveness, Expected value of perfect information, Partial expected value of perfect information, Quality-adjusted life-year, Incremental cost-effectiveness ratio

## Abstract

**Background:**

The surgical insertion of Ventilation Tubes (VTs) for the management of persistent bilateral Otitis Media with Effusion (OME) in children remains a contentious issue due to the varying opinions regarding the risks and benefits of this procedure. The aim of this study was to evaluate the economic impact of VTs insertion for the management of persistent bilateral OME in children, providing an additional perspective on the management of one of the commonest medical conditions of childhood.

**Methods:**

A decision-tree model was constructed to assess the cost-effectiveness of VTs strategy compared with the Hearing Aids (HAs) alone and HAs plus VTs strategies. The model used data from published sources, and assumed a 2-year time horizon and UK NHS perspective for costs. Outcomes were computed as Quality-Adjusted Life-Years (QALYs) by attaching a utility value to the total potential gains in Hearing Level in decibels (dBHL) over 12 and 24 months. Modelling uncertainty in the specification of decision-tree probabilities and QALYs was performed through Monte Carlo simulation. Expected Value of Perfect Information (EVPI) and partial EVPI (EVPPI) analyses were conducted to estimate the potential value of future research and uncertainty associated with the key parameters.

**Results:**

The VTs strategy was more effective and less costly when compared with the HAs plus VTs strategy, while the incremental cost-effectiveness ratio for the VTs strategy compared with the HAs strategy was £5,086 per QALY gained. At the willingness-to-pay threshold of £20,000 per QALY, the probability that the VTs strategy is likely to be more cost-effective was 0.58. The EVPI value at population level of around £9.5 million at the willingness-to-pay threshold of £20,000 indicated that future research in this area is potentially worthwhile, while the EVPPI analysis indicated considerable uncertainty surrounding the parameters used for computing the QALYs for which more precise estimates would be most valuable.

**Conclusions:**

The VTs strategy is a cost-effective option when compared with the HAs alone and HAs plus VTs strategies, but the need for additional information from future study is evident to inform this surgical treatment choice. Future studies of surgical and non-surgical treatment of OME in childhood should evaluate the economic impact of pertinent interventions to provide greater context.

## Background

Otitis Media with Effusion (OME) or glue ear is one of the commonest conditions of childhood. Approximately 80% of children suffer from OME at some point before reaching school age [1-3]. The presence of a thick mucoid or thin serous fluid within the middle ear space characterises OME, adversely affecting the efficiency of transfer of sound from the tympanic membrane (eardrum) to the hearing sensory organ (cochlea), leading to a conductive hearing loss. The middle ear space is normally an air filled chamber, across which sound is transferred by a chain of 3 ossicles (bones) to the cochlea in the inner ear. Hearing loss can lead to delayed speech and language development and adversely affect learning skills [4-6], and suspicion of hearing loss resulting from OME must be acted upon effectively [7]. OME can be resolved spontaneously, and this is reflected in the initial 3-month ‘watchful-waiting’ period advocated in the NICE (National Institute for Health and Care Excellence) Clinical Guideline 60 (CG60) developed by the National Collaborating Centre for Women’s and Children’s Health (NCCWCH) for the surgical management of OME in the United Kingdom (UK) [7]. However, OME is described to be a persistent problem if effusion, or fluid, within the middle ear space persists for at least 3 months. Children in whom OME persists after 3 months may require further action which can include surgery [7]. However, the interventions (preventive or curative) for OME are typically based on contemporary insights into the pathophysiology of the condition [8].

The commonest surgical treatment for OME is the insertion of Ventilation Tubes (VTs), also known as grommets or tympanostomy tubes, into the tympanic membrane [9,10]; a procedure that was first described by Armstrong [11] some 60 years ago. The insertion of VTs is one of the most commonly performed surgical procedures in the UK [12]. There is evidence both supporting and rejecting this surgical procedure, and hence, the use of VTs remains a contentious issue [1,6]. Furthermore, the decision to surgically insert VTs has not been assessed sufficiently well from a cost-effectiveness standpoint [12]. Amplification of hearing using conventional Hearing Aids (HAs) is a recognised alternative to the surgical intervention with VTs. However, it is important to understand that neither intervention is without associated risks, with both approaches ultimately reliant upon the natural resolution of the predisposing factors for OME, as can be expected in the majority of children with increasing age. The insertion of VTs involves the aspiration of the middle ear effusion, and is associated with a prompt improvement in natural hearing thresholds. Ventilation tube is designed to extrude naturally from the tympanic membrane after a given time, in anticipation that the predisposing factors for OME have resolved in the interim. The use of HAs also relies on the natural resolution of such predisposing factors, but instead of removing the cause of the conductive hearing loss, the intensity of the sound delivered to the ears is increased to compensate for the loss of energy across the fluid filled middle ear space. It is also described that parents with a child who has OME do not consider VTs and HAs as equal treatments [13].

Currently, there are no model-based economic evaluations that have directly compared the efficacy of HAs against VTs [7]. Whilst developing the NICE CG60 guideline [7], an economic evaluation was undertaken that explored whether the use of VTs was a cost-effective means of healthcare resources. Their economic model took the form of a decision-tree that compared the deterministic incremental costs and benefits of four strategies: *hearing aids*; *ventilation tubes*; *ventilation tubes plus adenoidectomy*; and *do nothing* for the management of children with persistent bilateral OME. However, the suggestions from their economic evaluation should be construed with a caution for the following reasons: (i) they used a 12-month follow-up time horizon for their model but the follow-up time duration should be 2 years or more for formal outcome measurements [14,15]. For instance, the harms that can be avoided by a surgical insertion usually occur in a reasonably distant future compared with the direct costs of surgery which incur much earlier; (ii) they allowed up to three surgical insertions to occur in a single year to a proportion of children which is unlikely to reflect actual clinical practice; (iii) they took a biased approach in which they assigned no gain in Quality-Adjusted Life-Year (QALY) associated with children in the hearing aids strategy; (iv) they assumed identical risk for subsequent VTs removal procedures, but a retrospective study suggested different risk for this [14]; (v) they did not conduct probabilistic sensitivity analysis, even though it has been suggested elsewhere [16]; and (vi) they did not analyse whether more evidence should be demanded to support the adoption decision. These points raise the question of how to come to a decision as to whether, or not, to recommend the surgical insertion of VTs on the basis of existing evidence.

It is common for the decision-makers to face uncertain choices when considering the adoption of healthcare interventions. To this end, the health economist argues that choices between alternative healthcare interventions should be made on the ground of which intervention generates more health benefit at a cheaper cost. However, when deciding as to whether to adopt a healthcare intervention, there is always a chance of making a wrong decision and there are costs associated with making the wrong decision. In this situation, the issue of whether, or not, to adopt an intervention should be weighed against whether more evidence should be demanded to support the adoption decision [17]. The value of acquiring additional information to inform a decision problem is based on the degree to which additional information will decrease this decision uncertainty. One of the suggested methods for reducing uncertainty is to conduct further research. For this purpose, the use of Value of Information (VOI) methods can conceptually and quantitatively offer a sound way to identify evidence gaps and prioritise future research [18,19], thereby supporting the decision-making as to whether additional research is justified. Claxton [20] some 15 years ago first proposed the use of VOI methods in the health economics literature. Since then, the use of VOI methods has been well-integrated and evolved in the context of the Health Technology Assessment Programme in the UK. Specifically, the key strength of VOI lies in estimating the maximum possible improvement in the net benefit associated with the decision that could be achieved, if the decision were to be made in a situation where there is perfect information rather than with the current level of information [21].

This study aimed to assess whether the management of persistent bilateral OME with VTs in ‘otherwise normal’ children is cost-effective and to perform VOI analyses in order to demonstrate the value for money from additional research. The use of VOI methods involved three necessary actions: (i) formation of decision-analytic model to describe the decision problem; (ii) stochastic illustration of the underlying decision uncertainties; and (iii) confirmation of the potential value of additional information from future study.

## Methods

**Figure 1:**
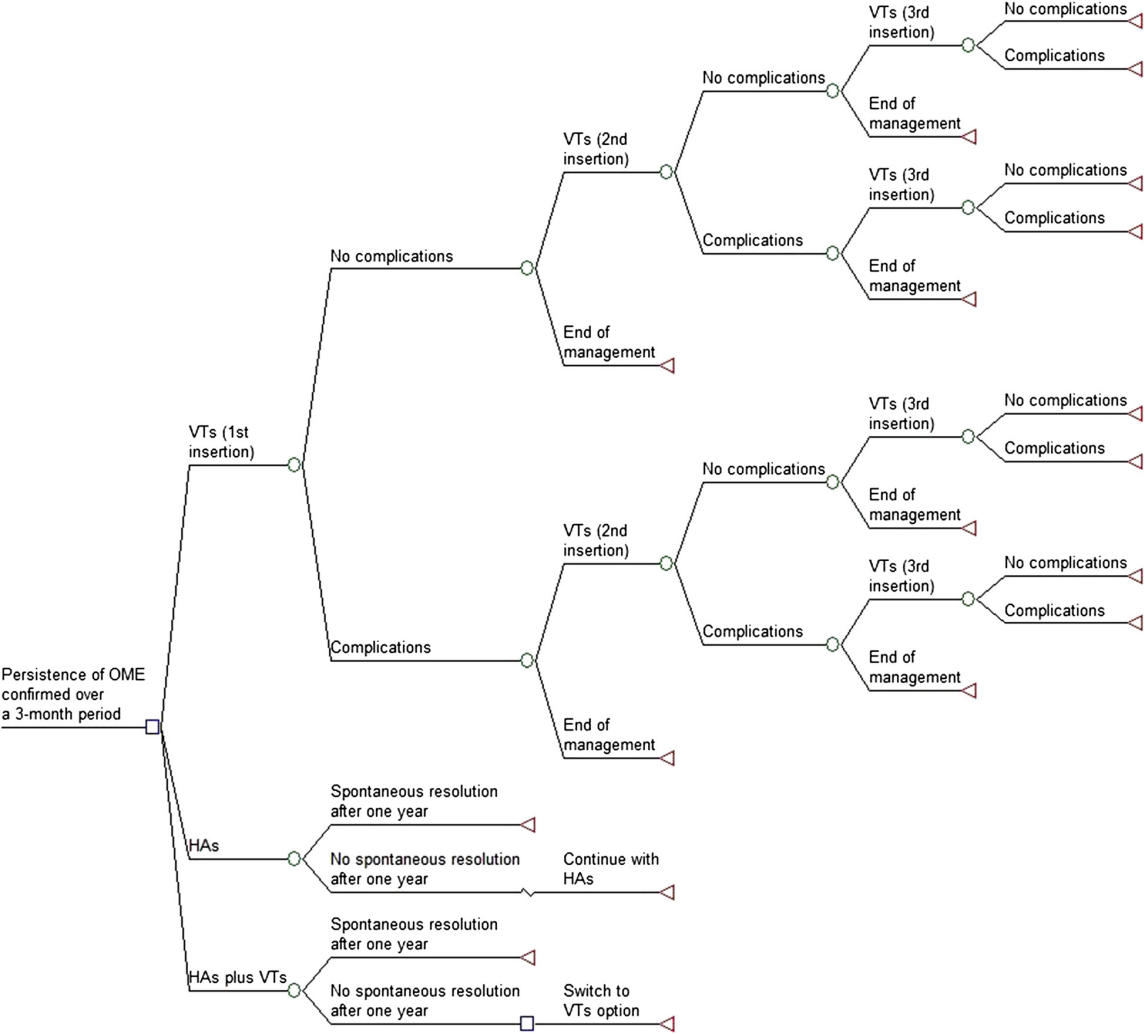
**The decision-tree diagram.** Figure footnote: The pathways of the HAs plus VTs strategy resemble that of the first 12 months of the HAs strategy and then of the first 12 months of the VTs strategy.

A decision-analytic model was developed to assess the cost-effectiveness of Ventilation Tubes (VTs) strategy compared with two alternative strategies: (i) Hearing Aids (HAs) alone; and (ii) HAs plus VTs, for the management of children with persistent bilateral OME. The decision-analytic model took the form of a decision-tree structure and a schematic of which is shown in Figure 1. This decision-tree structure has been slightly modified from the structure that was used to inform the NICE CG60 guideline on the surgical management of children with persistent bilateral OME [7]. A hypothetical cohort of 10,000 children under the age of 12 years was observed for a period of 24 months for each strategy. The model assumed a UK National Health Service (NHS) perspective for costs, and all costs and benefits incurred beyond 12 months were discounted at an annual rate of 3.5% [16]. The primary outcome of the model was to estimate the potential gain in Hearing Level in decibels (dBHL) associated with each strategy and express this in terms of QALY, which was estimated as a linear function by multiplying a utility value per unit increase in dBHL with the total potential gains in dBHL over two pre-defined time periods of 12 and 24 months. A simple example should serve to illustrate the concept. First, assume that the total potential hearing gains are 10 dBHL after 12 months and 8 dBHL after 24 months for a treatment option. Further, assume that the utility value per unit increase in dBHL is 0.01. Therefore, the QALY gain associated with this treatment option is: 10 × 0.01 + 8 × 0.01 = 0.18.

Utility is a value that characteristically represents a patient’s health-related quality-of-life on a scale between 0 (as bad as death) and 1 (perfect health) [22]. Subsequently, as suggested in the literature [23,24], cost against health benefit was explored through the estimation of Incremental Cost-Effectiveness Ratio (ICER), described as the difference in mean costs divided by the difference in mean QALYs between two interventions. An intervention that offers the lowest cost per QALY gained is typically considered to be cost-effective provided that the ICER remains within a willingness-to-pay threshold that a society is prepared to pay to gain a unit of health benefit (e.g. a QALY). Following the valuation proposed by NICE in UK, an ICER threshold of £20,000 per QALY gained has been used to judge whether an intervention is acceptable as an effective use of NHS resources [25]. Monte Carlo simulation was used to propagate the prior distributions assigned to model inputs and estimate the expected cost and QALY gain associated with each strategy. The three strategies compared and the assumptions made are now described.

### Ventilation Tubes (VTs) strategy

At the model starting point, this strategy assumed that children will require a surgical insertion of VTs once the persistence of bilateral OME is confirmed over a 3-month period. Given that fluid may accumulate in the middle ear space at a later date and VTs may extrude earlier than expected [1,7], some children are assumed to have a second insertion of VTs within the first year of treatment follow-up, and that some of these are to have a third insertion within the second year. The reason for having the third surgical insertion to take place within the second year of treatment follow-up was to avoid allowing multiple insertions in a 12-month period reflecting the most likely clinical practice. Repeated VTs insertions may increase the risk of long-term conductive hearing loss [26] and may increase the risk of scarring of the tympanic membrane. Thus, the model has limited the number of repeated surgeries per child to three [7,26]. It is assumed that inserted VTs will naturally extrude or fall out by 39 weeks [7].

Each surgical insertion was predicted to lead to a number of common post-operative complications including ear discharge (otorrhoea), formation of granulation tissue, and perforation of the tympanic membrane (see Table 1). These complications have been found to occur proportionately based on the meta-analysed estimates derived from a recently published systematic review of published case series and randomised studies [15]. It should be noted that the risk of tympanic membrane perforation has been predicted to be higher for subsequent surgeries [15]. The model has also explored the impact of the requirement to remove a child’s VTs, as might happen with recurrent infections [7,14]. This is not shown on the decision-tree, but was considered as part of the complications. Based on the suggestion that VTs insertion can help avoid the formation of cholesteatoma [7,10,27,28], no incidence of this risk was predicted for the children in this strategy. Likewise, formation of tympanosclerosis was not included as a complication of OME, reflecting the fact that this complication can occur in both ‘treated’ and ‘untreated’ cases, with the aetiology not being completely understood [29,30]. Post-operative risks such as severe bleeding, serious injury, or surgical mortality are not included in the model, as it is extremely unusual and unlikely for children to suffer any of these risks from an insertion of VT under modern anaesthesia [10,12,31].

**Table 1:** Probability data for VTs related post-operative complications

**Complication**	**Probability**	**Distribution for PSA**	**Source**	***Comments***
Ear discharge (otorrhoea)	0.262	Beta ~ (1439, 4052)	Kay et al. [15]	*It is assumed that children who suffer from this complication will need a course of medication from a GP (General Practitioner) visit*[7]
Formation of granulation tissue	0.042	Beta ~ (37, 850)	Kay et al. [15]	*It is assumed that children who suffer from this complication will need no surgery, but will need a course of medication from a GP visit*[7]
Perforation of tympanic membrane (1st insertion)	0.022	Beta ~ (178, 7929)	Kay et al. [15]	*It is assumed that children who suffer from this complication will need a surgery*[7]
Perforation of tympanic membrane (≥2 insertions)	0.166	Beta ~ (557, 2799)	Kay et al. [15]	

Children are assumed to require their first Ear, Nose and Throat (ENT) follow-up assessment within 6 weeks of each surgery and subsequent ENT assessments every 26 weeks thereafter, until the mean extrusion time of 39 weeks [7]. Children are also assumed to require on average 1.5 (1 or 2) audio-logical assessments of hearing threshold after each surgery [7]. The model assumed that a proportion of children who suffer from otorrhoea and/or granulation tissue formation will require making a visit to a General Practitioner (GP) for a course of antibiotics or eardrops. Table 1 shows the probabilities identified for VTs related post-operative complications, while Table 2 shows the probabilities identified for each aspect of the care pathway associated with the surgical insertion of VTs.

**Table 2:** Probability data for the VTs strategy

**Variable**	**Probability**	**Distribution for PSA**	**Source**	***Comments***
Removal of VTs (1st insertion)	0.072	Beta ~ (6, 77)	Phua et al. [14]	*Different risk has been predicted for subsequent removal procedures*[14]
Removal of VTs (≥2 insertions)	0.171	Beta ~ (7, 34)	Phua et al. [14]	
Re-insertion of 2nd VTs	0.25	Beta ~ (25, 75)	NCCWCH [7]	*Indifferent risk has been predicted for subsequent insertion procedures*[7]*, which is in line with the suggestion by Sheahan et al.*[49]*. Distribution for PSA is based on the report that 25 out of 100 children require 2 or more insertions of VTs*
Re-insertion of 3rd VTs	0.25	Beta ~ (25, 75)	NCCWCH [7]	

### Hearing Aids (HAs) strategy

This strategy assumed that children with persistent bilateral OME will require the use of HAs following the confirmation that bilateral OME has been persisted for 3 months. A meta-analysis has shown a spontaneous resolution rate of 30.8% when OME persisted in children for 12 months [32]; thus this spontaneous resolution rate was assumed to take place by the end of the first 12 months. However, children in whom OME had not spontaneously resolved at 12 months are assumed to continue with their HAs. Children are also assumed to have their first audio-logical assessment after 13 weeks from the model starting point and following audio-logical assessments every 26 weeks subsequently [7]. The initial costs of this strategy consist of the HAs devices, batteries and maintenance kit for HAs, HAs fitting, and ear-moulds. It is estimated that ear-moulds will need to be replaced every 13 weeks [7] and batteries will need to be replaced every 4 weeks [7]. Some of the costs will recur because the model assumed that 16.44% of children will accidentally break/lose their HAs over a period of 12 months [7].

Note that the middle ear effusion in this strategy is left untreated, with the amplification from HAs serving to improve the child’s access to sound. Untreated OME commonly leads to episodes of Acute Otitis Media (AOM) that requires treatment with an appropriate course of antibiotics [8,32,33]. AOM is the leading cause of clinic visits in the general childhood population [8] and children who are prescribed with antibiotics are usually encouraged to make more clinic visits for subsequent episodes [33]. Based on a reported meta-analysed value [32], an average of 2.8 episodes of AOM was predicted to occur each year for the children in this strategy. Therefore, it is assumed that every year children will make 2.8 GP visits for an appropriate course of antibiotics to manage emergent infection of AOM episodes [8,32,33]. AOM infection is not shown on the decision-tree diagram because this will have no impact on the predicted spontaneous resolution rate. Children are also assumed to make on average 1.5 (1 or 2) ENT visits [34,35] every year due to otorrhoea, granulation and/or AOM episodes. The only recorded HAs related complication has been reported to be non-adherence whereby a child would not use the device on a regular basis [34,36]. Although a very small proportion of children (9.1%) may not adhere to their HAs devices for the advised period of time each day [34], the model predicted that all children will adhere with their HAs. This will have no impact on the predicted spontaneous resolution rate, but will negligibly overestimate the QALY gain associated with the HAs strategy.

### HAs plus VTs strategy

This strategy assumed that in children with persistent OME confirmed over a 3-month period will initially be fitted with HAs. However, children who do not experience spontaneous resolution of OME by the end of the first 12 months are assumed to switch to the VTs strategy for the remainder of the follow-up period. The pathways of this strategy resemble that of the first 12 months of the HAs strategy (that is the first 12 months of this strategy) and then of the first 12 months of the VTs strategy (that is the last 12 months of this strategy).

### Model input parameters

The model input parameters were derived from different sources including a meta-analytic systematic review of published case series and randomised studies conducted by Kay et al. [15]. Recently, a Cochrane review was conducted by Lous et al. [1], but the relevant evidence that is available from this review was difficult to amalgamate due to the heterogeneous nature of identified studies covering a wide range of ages, a variety of outcome measures and small sample sizes. Therefore, the data used to populate the model were derived from the papers believed to have most direct relevance to the study population of interest. The model was supplemented with published expert opinion where data to populate the model was unavailable from other sources. The Probabilistic Sensitivity Analysis (PSA) (see Additional file 1) was performed in order to deal with uncertainty in the model input parameters. Distributions for parameters were selected on the basis of the nature of the parameter concerned. To conduct the simulations, the distributions were assigned to the model inputs to characterise the current uncertainty surrounding their values. The model was run for 10,000 simulations using Microsoft Excel^®^ in association with Visual Basic for Applications^®^. For each simulation, the value of each variable was sampled at random from the distributions specified. The mean expected costs and QALYs were estimated by averaging the outputs of all simulations. To calculate the QALY gain associated with each strategy, a utility value of 0.00874 (95% CI: 0.005 to 0.012) per unit increase in dBHL was attached to the total gains in dBHL identified for each strategy over two specified time periods (see Table 3). This utility value was published in the NICE CG60 guideline [7] and analysed by an expert upon receiving individual-patient data from Kubba [37] for children with a median age of 5 years. Table 3 shows the model inputs in terms of average gain in dBHL, while Table 4 summarises the resources and unit costs used for each strategy.

**Table 3:** dBHL parameters

**Strategy**	**dBHL**		**Distribution for PSA**	**Source**	***Comments***
	**After 1 year**	**After 2 years**			
VTs	13.06	12.24	Normal ~ (13.06, 9.49) Normal ~ (12.24, 9.1)	Maw & Bawden [43]	*Normal distributions were used as part of the PSA to reflect the likelihood of an increased or decreased unit in dBHL*
HAs	4.88	7.57	Normal ~ (4.88, 11.11) Normal ~ (7.57, 12.76)	Maw & Bawden [43]	

**Table 4:** Resource use and unit cost data (costs are shown in pound sterling for the year 2010–11)

**Resource**	**Mean unit cost**	**Distribution for PSA**^**a**^	**Source**	***Comments***
Insertion of VTs	£891	Gamma ~ (130.45, 6.83)	NSRC1^b^ 2010–11 (HRG^c^ code CZ08T; day case)	*Minor ear procedures for ≤ 18 years old children through tympanic membrane. One procedure is assumed to insert two VTs for each child*
Tympanoplasty	£1,831	Gamma ~ (100.11, 18.29)	NSRC1^b^ 2010–11 (HRG^c^ code CZ10U; day case)	*Major ear procedures for ≤ 18 years old children. One procedure is assumed*
Removal of VTs	£891	Gamma ~ (130.45, 6.83)	NSRC1^b^ 2010–11 (HRG^c^ code CZ08T; day case)	*Minor ear procedures for ≤ 18 years old children through tympanic membrane. One procedure is assumed to remove one or two VTs for each child*
HAs	£80	Fixed	GDG^d^ estimate [7] was inflated based on the HCHS^e^ index 2010–11 (PSSRU^f^)	*Each child needs two HAs due to bilateral condition*
Ear-moulds	£17	Fixed	GDG^d^ estimate [7] was inflated based on the HCHS^e^ index 2010–11 (PSSRU^f^)	*Each child needs two ear-moulds in every 13 weeks*[7]
Maintenance kit for HAs	£20.88	Fixed	http://www.connevans.co.uk	*Cost of buying a maintenance kit for the HAs. This cost incurs once*
Batteries for HAs	£0.49	Fixed	http://www.hearing-aid-batteries.org.uk	*Batteries need to be replaced every 4 weeks*[7]
HAs fitting	£76	Gamma ~ (71.03, 1.07)	NHS Reference Costs (2005–06) (Service code AS1FA) was inflated based on the HCHS^e^ index 2010–11 (PSSRU^f^)	*Fitting of HAs in an audiology department. One procedure is assumed to fit two HAs for each child due to bilateral OME. This cost incurs again when a child looses or breaks his/her HAs*[7]
Medication	£11	Fixed	GDG^d^ estimate [7] was inflated based on the HCHS^e^ index 2010–11 (PSSRU^f^)	*Cost for a course of antibiotic or eardrop due to otorrhoea, granulation and/or AOM. One course is assumed*[7]
GP visit	£41	Gamma ~ (341.67, 0.12)	PSSRU^f^ (unit costs of health and social care 2010–11)	*GP visit fee for otorrhoea, granulations and/or AOM. Children who suffer from otorrhoea and/or granulation (see Table*1*) are assumed to make the visit. On the other hand, 2.8 visits per year are assumed for children in the HAs strategy*[8,32,33]
Audio-logical assessment	£48	Gamma ~ (64, 0.75)	NHS Reference Costs (2005–06) (Service code AS1FU) was inflated based on the HCHS^e^ index 2010–11 (PSSRU^f^)	*One or two visit(s) following a surgery*[7]*. One visit within 13 weeks and subsequent visits every 26 weeks thereafter for the HAs strategy*[7]
ENT visit	£91.72	Gamma ~ (84.14, 1.09)	NSRC1^b^ 2010–11 (Service code 120)	*One visit within 6 weeks of a surgery and subsequent visits every 26 weeks thereafter until the time of extrusion*[7]*. One or two visit(s) per year*[34,35]*for the HAs strategy*

^a^Cost data were sampled between the reported lower and upper quartile values.

^b^NHS Reference Costs 2010–11 (Appendix NSRC1).

^c^Healthcare Resource Groups.

^d^Guideline Development Group [7].

^e^Hospital and Community Health Services.

^f^Personal Social Services Research Unit.

### Value of Information (VOI) analyses

The outputs of the simulations were also used to conduct VOI analyses [21], and the first step of the exploration of VOI involved the estimation of a value known as the Expected Value of Perfect Information (EVPI). As shown in Equation 1, EVPI is the difference between the expected value of a decision with perfect information and the expected value of a decision with current information

(1) $$\mathrm{EVPI}=E_{\theta }\max_{j}\mathrm{NB}\left(j,\theta \right)-\max_{j}E_{\theta }\mathrm{NB}\left(j,\theta \right)$$

where E_θ_*max*_j_NB(j,θ) corresponds to the maximum expected value of the decision with perfect information and *max*_j_E_θ_NB(j,θ) corresponds to the maximum expected value with current information. The NB (Net Benefit) associated with each treatment option was calculated in monetary terms using the statistic: NB = λ × QALY – cost, where λ represents a willingness-to-pay threshold amount that one is prepared to pay to gain one unit of QALY. For example, if the willingness-to-pay threshold of a QALY is £20,000, then for a treatment that provides 2.0 QALYs for a cost of £10,000; the NB is: £20,000 × 2 – £10,000 = £30,000. That is, £30,000 is the average NB of introducing this treatment.

Using Equation 2, EVPI was then estimated across the population of future OME affected children for whom the decision is relevant

(2) $$\mathrm{pEVPI}=\mathrm{EVPI}\;\sum_{t=1}^{T}\frac{I_{t}}{{\left(1+r\right)}^{t}}$$

where I_t_ = occurrence of OME relevant to VTs, T = effective lifetime of VTs, and r = discount rate. The pEVPI (EVPI at population level) estimate was based on an assumption that every year 25,000 children in the UK would qualify for the surgical insertion of VTs [38] that would continue for at least another 5 years given its existence for almost last 60 years [11]. This yielded an effective total population of approximately 116,827 discounted at a rate of 3.5% over 5 years.

Based on the advice from literature [19,39], the basic method for estimating EVPI was further extended to identify the most important parameters for which more precise estimates would be most valuable. This method of analysis is known as the Expected Value of Partial Perfect Information (EVPPI), which is estimated using Equation 3

(3) $${\mathrm{EVPPI}}_{\vartheta }=E_{\vartheta }\max_{j}E_{\varphi /\vartheta }\mathrm{NB}\left(j,\varphi ,\vartheta \right)-\max_{j}E_{\theta }\mathrm{NB}\left(j,\theta \right)$$

where *ϑ* represents parameter of interest, *φ* represents other uncertainties, *θ* represents all parameters, E_*ϑ*_*max*_j_E_φ/*ϑ*_NB(j,φ,*ϑ*) corresponds to the maximum expected value with perfect information for parameter *ϑ*, and *max*_j_E_θ_NB(j,θ) corresponds to the maximum expected value of current information for all parameters *θ*. The EVPPI analysis was conducted on a group of parameters including the utility gain per unit increase in dBHL and the total gain in dBHL, because a priori this would impact the expected QALY gain associated with each strategy.

## Results

**Figure 2:**
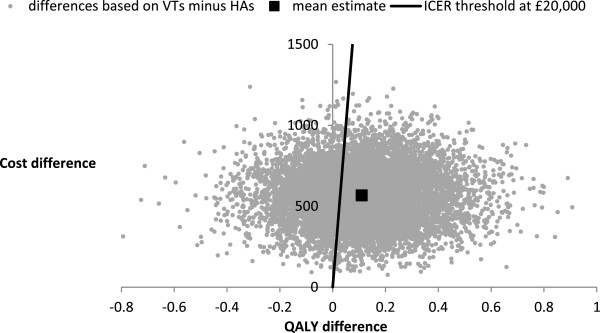
Scatter-plot showing the mean differences in costs and in QALYs from the PSA.

**Figure 3:**
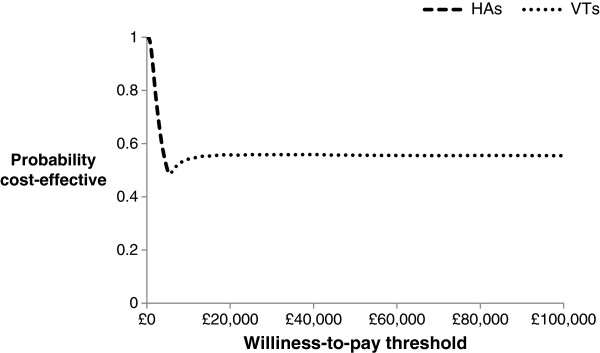
CEAFs for the strategies compared.

The results of the Probabilistic Sensitivity Analysis (PSA) are presented in terms of costs and outcomes per child in Table 5, which indicates that the VTs strategy is associated with the lowest cost per QALY gained. The VTs strategy ‘strongly dominated’ the HAs plus VTs strategy because the VTs strategy was more effective and less costly comparing with the HAs plus VTs strategy. The ICER for the VTs strategy compared with the HAs strategy was £5,086 per QALY gained. This ICER value is well below £20,000 that is viewed by the NICE to be a realistic threshold for willingness-to-pay for an additional QALY gained [25]. At the willingness-to-pay threshold of £20,000, the VTs strategy was found to offer the highest net benefit. Typically, a strategy that offers the highest net benefit is considered to be the preferred option [21].Figure 2 shows the scatter-plot of mean differences in costs and in QALYs from the PSA using 10,000 simulations. Given that the VTs strategy ‘strongly dominated’ the HAs plus VTs strategy, the data in Figure 2 were generated by comparing the VTs strategy with the HAs strategy. With the positive mean differences in costs and in QALYs, some 90% of the simulated data indicated that the VTs strategy would be cost-effective compared with the HAs strategy under the willingness-to-pay threshold of £20,000 per QALY gained. Figure 3 shows the Cost-Effectiveness Acceptability Frontiers (CEAFs) across different willingness-to-pay thresholds. At the willingness-to-pay threshold of £20,000, the probability that the VTs strategy is likely to be more cost-effective was 0.58. The CEAFs show that the VTs strategy is most likely to be cost-effective beyond the willingness-to-pay threshold of £5,500 per QALY gained.

**Table 5:** Various results from the PSA

**Strategy**	**Cost**	**QALY gained**	**Cost per QALY gained**	**ICER**	**Net benefit (willingness-to-pay threshold of £20,000)**
HAs	£1,237	0.107	£11,593		£909
VTs	£1,801	0.218	£8,277	£5,086 (VTs vs. HAs)	£2,544
HAs plus VTs	£2,498	0.139	£17,971		£285

**Figure 4:**
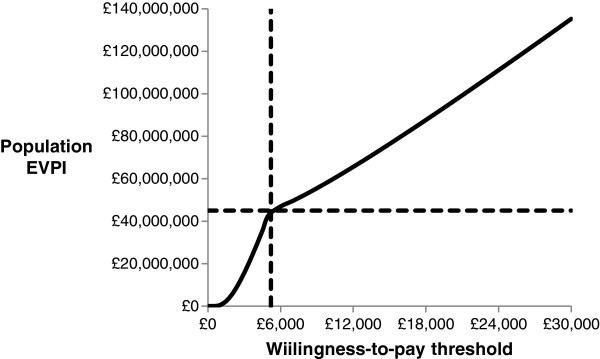
Expected value of perfect information at the population level.

The EVPI analysis offered an upper bound of the potential value of conducting future research to reduce underlying decision uncertainty over whether the surgical insertion of VTs is cost-effective than its comparators. The EVPI values at individual and population levels for a range of different willingness-to-pay thresholds are presented in Table 6. The pEVPI estimation was based on an assumed effective children population of 116,827 in UK who were thought to receive VTs over a decision horizon of 5 years.Figure 4 shows the pEVPI values at different willingness-to-pay thresholds. The potential value of additional information was found to be high beyond the willingness-to-pay threshold of £5,500. For instance, at the willingness-to-pay threshold of £5,500, the VTs strategy would accrue the maximum potential value of around £4.6 million at population level by acquiring additional information from future study. The pEVPI value of around £9.5 million at the willingness-to-pay threshold of £20,000 indicated that this would potentially be worthwhile to conduct future research. The pEVPI values were found to be increasing as the willingness-to-pay thresholds were increasing.

**Table 6:** EVPI values at individual and population levels at different willingness-to-pay-thresholds

**Willingness-to-pay threshold**	**EVPI at individual level**	**EVPI at population level**
£5,000	£366	£42,758,675
£10,000	£503	£58,763,971
£15,000	£654	£76,404,845
£20,000	£817	£95,447,643
£25,000	£986	£115,191,402
£30,000	£1,158	£135,285,643

The EVPPI value associated with the QALY gain arising from the utility gain per unit increase in dBHL and the total gains in dBHL over 12 and 24 months was investigated. Following the advice of Brennan et al. [39], a total of 100,000 simulations (100 simulations in the outer loop and 1,000 simulations in the inner loop) were used to estimate the EVPPI value at population level of around £7.4 million at the willingness-to-pay threshold of £20,000. This indicates that the QALY gain associated with each strategy entails significant uncertainty. Therefore, if future research is to be conducted, it should then prioritise looking into these important parameters for which more precise estimates from future research would be most valuable.

## Discussion

This study assessed the impact of surgical insertion of VTs compared with alternative options such as HAs alone and HAs plus VTs. The use of HAs as an alternative option was chosen for the following key reasons: (i) some parents and their children prefer non-surgical intervention such as HAs [7,13,34,36]; (ii) HAs are a well-established intervention and are considered to be a viable alternative to VTs in the management of persistent OME [7,13]; (iii) parents do not consider HAs and VTs as equal treatments [13]; and (iv) there are no good quality model-based economic evaluations that compared the efficacy of HAs against VTs [7,12,40]. The inclusion of HAs plus VTs as another alternative management strategy reflects current literature [13,34,36]. ‘Extended period of watchful-waiting’ or ‘do-nothing’ was not considered as an alternative management option because of the following reasons: (i) children in whom bilateral OME persists over 3 months show a progressively low resolution rate [32,41,42]; (ii) the ‘do-nothing’ option may reduce some potential costs, but the overall use of resources may in fact increase due to the requirement for more regular follow-up(s) [7,12]; (iii) leaving OME untreated is not an advisable option in persistent bilateral cases because this may amplify the problem of conductive hearing loss [43]; (iv) it increases the pressure on clinicians to prescribe antibiotics [44]; (v) it encourages children and their parents to make regular GP or hospital visits [33]; and (vi) use of VTs or HAs are believed to quickly improve overall hearing performance [45].

The PSA was conducted because decision-makers who review healthcare interventions are usually keen to comprehend the level of uncertainty [46]. This is also viewed as a measure of the basic quality of any published model-based economic evaluation [16,47]. A key advantage of the PSA was that it facilitated the VOI analyses, which are increasingly being applied for future research prioritisation within the evidence-based practice centre programme [48]. Lessons were drawn from previously published studies regarding the wider application of the VOI methods, and as part of this study, EVPI and EVPPI methods were conducted to determine whether more evidence would potentially augment the relative worth of the surgical insertion of VTs. Both of these methods are recognised as a rational methodological extension of the PSA [18,20]. The EVPI analysis was important in deciding whether the value of undertaking future research is potentially worthwhile, while EVPPI extended this to provide the breakdown on key uncertain parameters. Although other methods such as confidence intervals or Bayesian credible intervals could have been used, the feasibility and implications of using VOI methods for informing future research prioritisation process are highly recommended for dealing underlying decision uncertainty [18,20,39].The results of this study suggest that the surgical insertion of VTs will cost more than the HAs option, but result in additional benefits over the willingness-to-pay threshold of £5,500. The CEAFs (see Figure 3) showed that there is about 58% probability to achieve an additional gain in QALY at an additional cost of £20,000 for every child who is managed with VTs. The results of the PSA revealed a considerable amount of uncertainty; nevertheless the management of the condition with VTs generated more expected net benefit to compensate the additional expenditure incurred. The pEVPI value of around £9.5 million at the willingness-to-pay threshold of £20,000 suggested that the value of future research in this area of research would potentially be worthwhile provided that the total cost of undertaking future research does not exceed £9.5 million. However, whether this research should be undertaken will depend upon the cost of the study into the key uncertain parameters and the exact threshold under which the decision is to be made. The main uncertainty arose from the use of parameters that drove the estimation of QALY associated with each strategy. The EVPPI analysis suggested that future research to further evaluate the utility gain per unit increase in dBHL and the total gain in dBHL parameters would be most valuable.

A caveat is that the use of the utility value of 0.00874 for each unit increase in dBHL was obtained from a source [7], which could not be verified. It was not clear if this gain in utility is directly relevant to OME affected children, but OME is universally associated with temporary loss of hearing. Mathematically, the use of this utility increment cannot favour any specific strategy in terms of effectiveness comparison because the same utility increment was used across all strategies for each unit increase in dBHL. Although this is a simplifying statement, there were no reliable data that could differentiate the outcomes with respect to hearing problem. Another caveat is that the distributions around the cost parameters (see Table 4) used in the PSA were estimated based on the reported lower and upper quartile values. This analysis was based on a hypothetical cohort of 10,000 ‘otherwise normal’ children aged 12 years or under. This age cut-off was selected because of the following key reasons: (i) the ear problems appear to settle in children over the age of 12 years [49]; (ii) the condition is most prevalent under the age of 12 years [1]; and (iii) there is a shortage of epidemiological data for other age groups. The time horizon for the model was 24 months which is suggested to be a reasonable follow-up period for formal outcome measurement [7,14]. Interpretation of this analysis should be undertaken with caution, as with no definitive guidelines identified for the treatment of OME in children, the clinical pathway used to structure the economic evaluation was developed using assumptions based on available published evidence. As a consequence, the economic evaluation was regarded investigative and not as a validation for any changes into routine clinical practice at present.

## Conclusions

Childhood hearing loss resulting from OME is well-documented. This study has assessed the decision to surgically insert VTs from a cost-effectiveness standpoint. Based on the assumptions used in this analysis, the surgical insertion of VTs for the management of persistent bilateral OME in children is most likely to be a cost-effective option, but the need for additional information from future study is evident to inform this treatment choice. This study has also shown that the value of additional information is potentially worthwhile and identified the QALY gain arising from the utility gain per unit increase in dBHL and the total gain in dBHL as the key uncertain parameters. This analysis should be reassessed once adequate clinical effectiveness data become available. The results presented here should not be considered as an option for all age groups; thus further research is required to identify sub-groups of children likely to benefit most from the surgical insertion of VTs. To provide greater context, future studies of surgical and non-surgical treatment of OME in childhood should evaluate the economic impact of relevant interventions.

## Competing interests

The authors declare that they have no competing interests.

## Authors’ contributions

SM, AS and IB all contributed to this paper. SM wrote the first draft of the paper and all authors commented on it and subsequent drafts. All authors meet the guidelines for authorship of the International Committee of Medical Journal Editors. All authors read and approved the final manuscript.

## Pre-publication history

The pre-publication history for this paper can be accessed here:

http://www.biomedcentral.com/1472-6963/14/253/prepub

## Supplementary Material

Additional file 1 Probabilistic Sensitivity Analysis (PSA). Click here for file
