# Supplementary material for: Economic evaluation of surgical insertion of ventilation tubes for the management of persistent bilateral otitis media with effusion in children
Source: BMC Health Serv Res. 2014 Jun 13;14:253. doi: 10.1186/1472-6963-14-253 (PMC4112653; doi:10.1186/1472-6963-14-253)
Supplement: Additional file 1 — Probabilistic Sensitivity Analysis (PSA). [file 1472-6963-14-253-S1.docx]

Appendix 1 Probabilistic Sensitivity Analysis (PSA)

Following the advice of NICE [16], the PSA was run in that all input parameters were entered as probability distributions to reflect their imprecision and Monte Carlo simulation was used to reflect this uncertainty in the model’s outputs. The beta distribution was used for the probability parameters (see Tables 1 and 2) since these values are restricted between zero and one, the normal distribution was used for the gain in dBHL parameters (see Table 3) reflecting the fact that the level of hearing can increase or decrease during the recovery period, and the gamma distribution was used for the resource use and cost parameters (see Table 4) since these values are constrained to be non-negative. The beta distribution has two parameters: first one corresponds to the ‘number of events’ and second one corresponds to the ‘number of non-events’. The normal distribution has two parameters: first one corresponds to the ‘mean’ and second one corresponds to the ‘variance’. The gamma distribution is parameterised by two parameters, which are expressed as functions of the mean and variance of the distribution. In the PSA, prospective AOM episodes were sampled from the distribution labelled as Normal~(2.8, 0.3) [32] and the rate of spontaneous resolution was sampled from Beta~(61, 137) [32]. VTs should fall out between 26 and 52 weeks [7], and hence, the distribution labelled as Normal~(39, 2.93) was used to sample the data related to time to extrusion. The utility gain per unit increase in dBHL was sampled from the distribution labelled as Gamma~(24.38, 0.0004), which are estimated from the mean and variance reported earlier. Under the assumption of a constant hazard, the probability of breakage/loss of HAs was sampled based on the estimates that 25% of children break/lose their HAs over a 21-month period [7].
